# Ultrasonography screening of hepatic cystic echinococcosis in sheep flocks used for evaluating control progress in a remote mountain area of Hejing County, Xinjiang

**DOI:** 10.1186/s12917-024-04074-z

**Published:** 2024-05-17

**Authors:** Xinwei Qi, Tao Song, Zhao Li, Tao Jiang, Zhuangzhi Zhang, Chuanchuan Wu, Gang Guo, Jian Dong, Wubulitalifu Dawuti, Jingrui Dou, Jun Li, Hao Wen, Wenbao Zhang

**Affiliations:** 1 State Key Laboratory of Pathogenesis, Prevention and Treatment of High Incidence Diseases in Central Asia, Clinical Medical Research Institute, The First Affiliated Hospital of Xinjiang Medical University, No. 137 South Liyushan Road, Urumqi, 830054 Xinjiang China; 2 Veterinary Research Institute, Xinjiang Academy of Animal Sciences, No. 468 Alishan Road, Urumqi, 830011 Xinjiang China; 3 Animal Center, Xinjiang Medical University, No. 393 Xinyi Road, Urumqi, 830000 Xinjiang China; 4 Basic Medical College, Xinjiang Medical University, No. 567 North Shangde Road, Urumqi, 830039 Xinjiang China; 5 Suzhou Center for Disease Control and Prevention, No. 72 Sanxiang Road, Suzhou, 215004 Jiangsu China

**Keywords:** Ultrasonography, Cystic echinococcosis, *Echinococcus granulosus*, Sheep flocks, Active cysts

## Abstract

**Background:**

Although ultrasonography (US) has been widely used in the diagnosis of human diseases to monitor the progress of cystic echinococcosis (CE) control, the screening method for hepatic CE in sheep flocks requires adjustment. In this study, we used a US scanner to screen sheep flocks and evaluated the efficacy of dosing dogs once a year with praziquantel for 7 years from 2014 to 2021.

**Methods:**

All sheep in the three flocks were screened using an ultrasound scanner in 2014 and compared with the prevalence of infection in 2021 in Bayinbuluke, Xinjiang, China. Sheep age was determined using incisor teeth. Cyst activity and calcification were determined using US images. The dogs were dewormed with praziquantel once a year to control echinococcosis in the community.

**Results:**

Three flocks had 968 sheep in 2014, with 13.22%, 22.62%, 18.7%, 27.27%, 11.88%, and 6.3% of sheep aged 1, 2, 3, 4, 5, and ≥ 6 years old, respectively. US scanning revealed that the overall CE prevalence was 38.43% (372/968), with active cysts and calcified cysts present in 9.40% (91/968) and 29.02% (281/968) of the sheep, respectively. For the young sheep aged 1 and 2 years, the prevalence of active and calcified cysts was: 1.56% and 0.91%, and 10.94% and 18.72%, respectively. Approximately 15.15% and 16.52% of the 4- and 5-year-old sheep, respectively, harbored active cysts. There was no significant difference in the infection rates of sheep between 2014 and 2021 (*P* > 0.05).

**Conclusions:**

US is a practical tool for the field screening of CE in sheep flocks. One-third of the sheep population in the flocks was 1–2 years old, and these sheep played a very limited role in CE transmission, as most of the cysts were calcified. Old sheep, especially culled aged sheep, play a key role in the transmission of CE. Dosing dogs once a year did not affect echinococcosis control.

**Supplementary Information:**

The online version contains supplementary material available at 10.1186/s12917-024-04074-z.

## Background

Cystic echinococcosis (CE), caused by the dog tapeworm *Echinococcus granulosus sensu stricto*, is a zoonosis that causes considerable global health problems and economic losses [1–3]. Highly endemic areas include Western China, Central Asia, countries around the Mediterranean region, North Africa, and South America [4]. According to current estimates of the global burden, CE accounts for 285,500 disability-adjusted life years and causes approximately USD 2 billion in annual economic losses in the livestock industry [1, 5, 6]. Although CE is a chronic disease with low mortality (2–4%), it may cause severe health conditions and target organ dysfunction if proper care management is not provided [1].

The life cycle of *E. granulosus ss* requires two mammalian hosts. Adult worms inhabit the small intestine of canids, normally dogs, as definitive hosts, whereas the larval cyst (metacestode stage) develops in the internal organs (mainly the liver) of intermediate hosts [7]. Sheep are the intermediate hosts most adapted to this parasite and play a key role in the transmission of CE [7, 8]. Only sheep with active cysts, especially cysts containing protoscoleces (PSCs), play a role in the transmission of CE. Normally, epidemiological data on CE in sheep are collected from slaughterhouses. However, as the culled sheep slaughtered in slaughterhouses are normally from different areas (e.g., approximately 20% of sheep slaughtered in Urumqi slaughterhouses are from Qinghai and Gansu provinces, personal communication with Prof. Hao Wen, First Affiliated Hospital of Xinjiang Medical University), the data may not truly reveal the epidemiological situation in the target areas. In addition, as most slaughtered sheep are either young (lambs) or old (ewes), aged more than 5 years, the slaughterhouse survey may have biased the epidemiological situation. In addition to the control program, an easy and non-killing method is urgently required to monitor the progress of CE control, and ultrasonography (US) has been suggested for this purpose.

US has been widely used for CE diagnosis in clinical practice [1, 9]. The World Health Organization (WHO) published guidelines for detecting echinococcal infections in patients using the US technique [10]. Several studies have shown that US can be used to screen for CE in sheep [11, 12]. Dore et al. (2014) showed that US has a sensitivity of 88.7% and a specificity of 75.9% compared to post-mortem examination as the gold standard [11]. However, this method needs to be adopted in remote villages or sheep farms to monitor progress in the control of CE.

The present study aimed to use a portable US scanner to screen all sheep in three flocks in a remote mountain pasture community and to evaluate the control program for 7 years. We showed that CE in sheep is highly endemic in the study area. However, most cysts were calcified. We also showed that the percentage of active *E. granulosus* cysts was low in the flocks, which may have affected the accurate calculation of the dynamic transmission of CE.

## Results

### Sheep aged less than 2 years were the predominant age group in the flocks

**Fig. 1:**
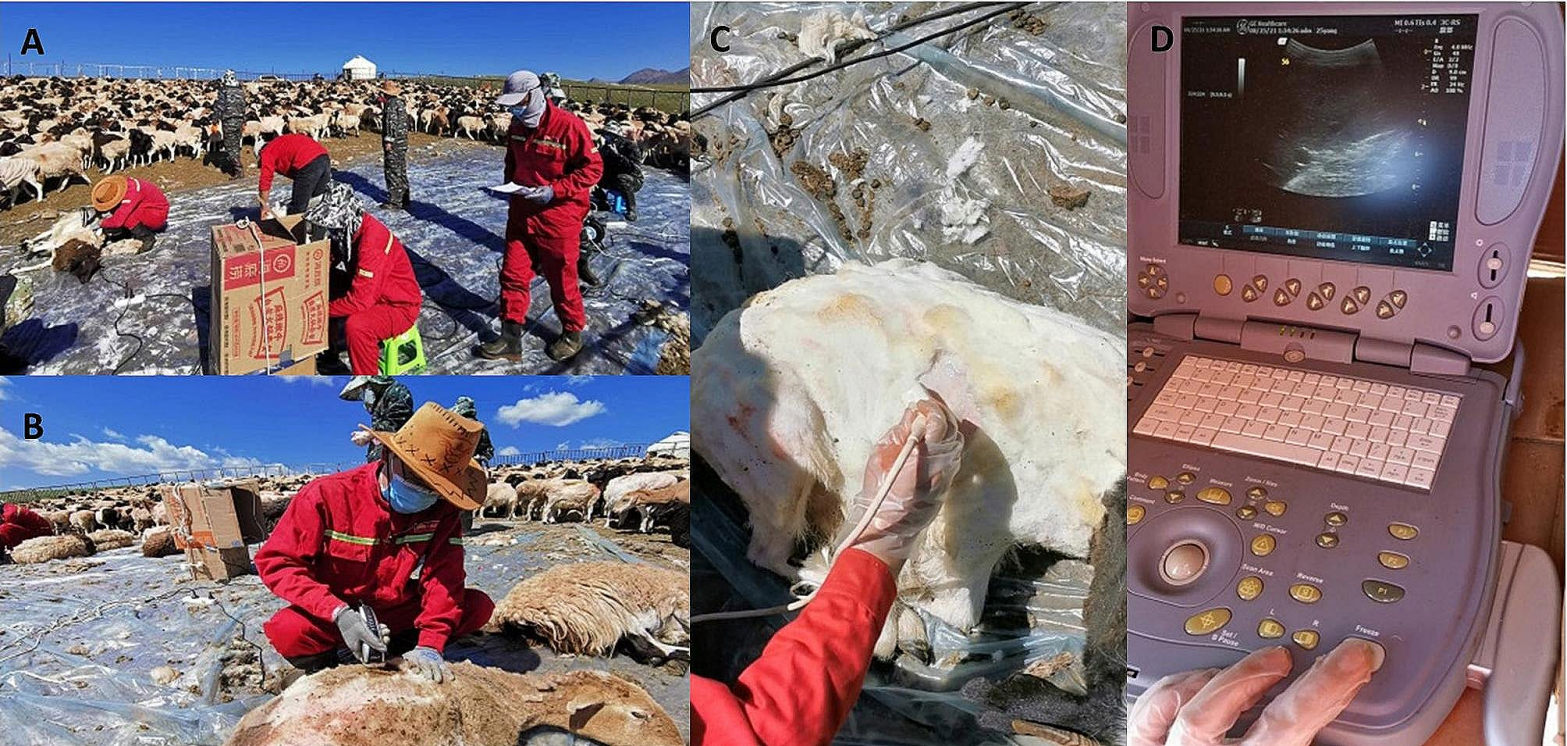
Field detection of cystic echinococcosis in sheep using ultrasonography. **A**, Test field in a mountain sheep farm; **B**, Shaving a window area in the right thorax of a sheep after shearing; **C**, Probing with a convex ultrasound transducer probe; **D**, Screening and recording with a GE book model ultrasound scanner

**Fig. 2:**
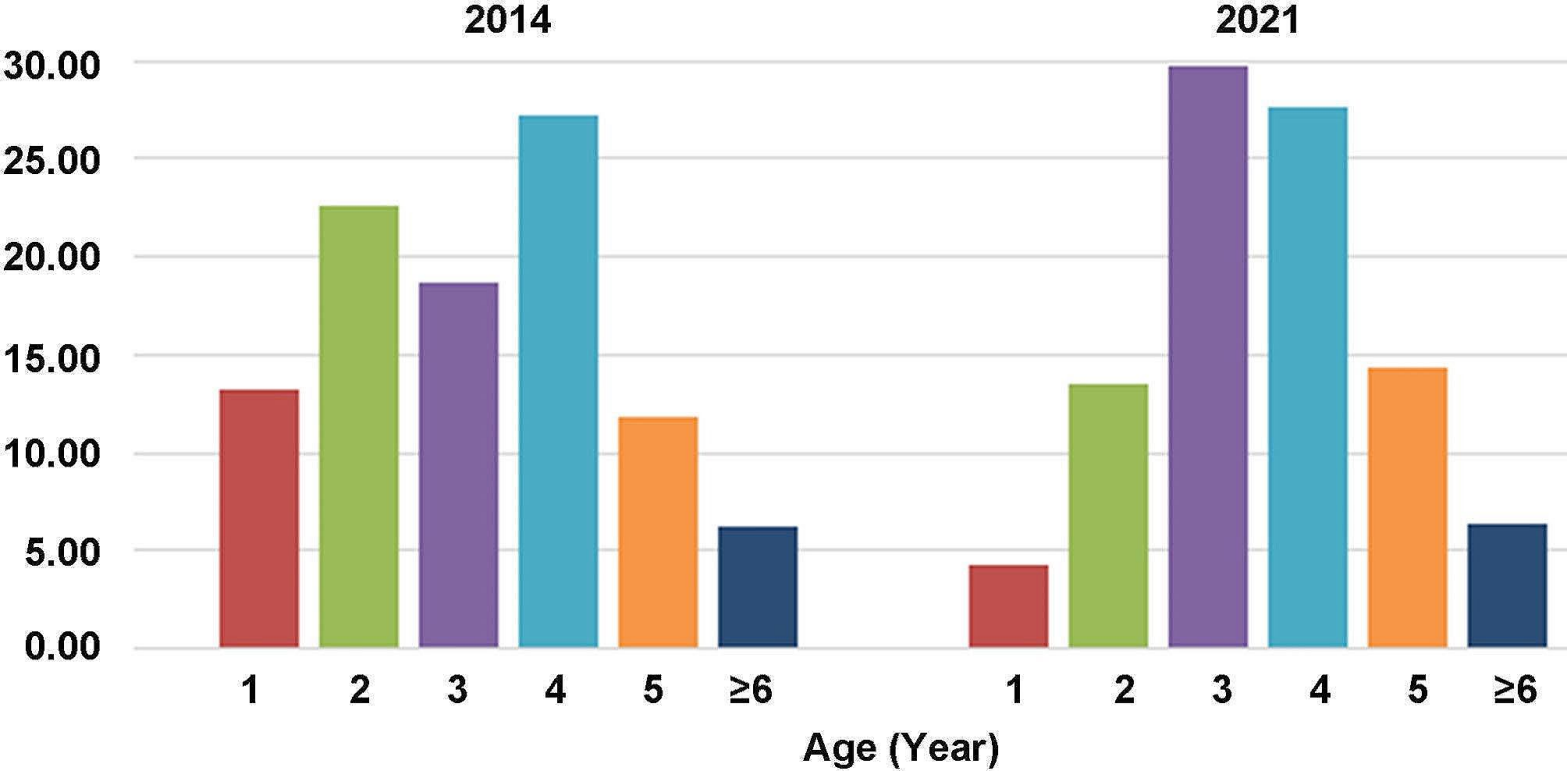
Age structure of whole sheep flocks in 2014 and randomly selected sheep in 2021

Figure 1 shows the usefulness and procedure of using the US book model to detect sheep liver lesions on mountain pasture farms. Table 1; Fig. 2 show the age structure of whole sheep flocks and the infectious prevalence in each age group in 2014. A total of 968 ewes between 1 and 8 years old (3.24 ± 1.51) from three farms were examined in 2014, excluding 226 newborn female lambs aged less than 4 months. Considering that the sheep age structure may impact the dynamic transmission of CE, we drew an age structure for these ewes (Fig. 2; Table 1, Tables S1–S5), which showed that 35.84% (347/968) of the checked sheep were aged 1–2 years. If these lambs were included, sheep aged less than 2 years comprised 47.99% (573/1194) of the total sheep population in these flocks. Sheep aged 3, 4, 5, and ≥ 6 years accounted for 18.70%, 27.27%, 11.88%, and 6.3% of the sheep population, respectively (Table 1; Fig. 2, and Tables S1–S3). In 2021, #2 flock sheep were sold. In the two existing flocks, 469 sheep aged between 1 and 7 years (3.58 ± 1.29) were randomly selected from 994 ewes, excluding 202 lambs aged less than 4 months(Table 1 and Tables S4–S5.)

**Table 1:** Age groups and infectious status in three flocks in 2014 and two flocks in 2021 in Bayinbuluke

Age	Number of sheep (%*)		Positive (%)		Active cysts (%)		Calcified (%)	
	2014	2021	2014	2021	2014	2021	2014	2021
1	128 (13.22%)	21 (4.48%)	16 (12.50%)	6 (28.57%)	2 (1.56%)	3 (14.29%)	14 (10.94%)	3 (14.29%)
2	219 (22.62%)	66 (14.07%)	43 (19.63%)	8 (12.12%)	2 (0.91%)	4 (3.03%)	41 (18.72%)	4 (6.06%)
3	181 (18.70%)	146 (31.13%)	70 (38.67%)	46 (31.51%)	20 (11.05%)	12 (8.22%)	50 (27.62%)	34 (23.29%)
4	264 (27.27%)	135 (28.78%)	144 (54.55%)	72 (53.33%)	40 (15.15%)	19 (14.07%)	104 (37.50%)	53 (39.26%)
5	115 (11.88%)	70 (14.93%)	68 (59.13%)	39 (55.71%)	19 (16.52%)	20 (28.57%)	49 (39.13%)	19 (27.14%)
> 6	61 (6.30%)	31 (6.61%)	31 (50.82%)	22 (70.97%)	8 (13.11%)	16 (51.61%)	23 (36.07%)	6 (19.35%)
Total	968	469	372 (38.43%)	193 (41.15%)	91 (9.40%)	74 (15.78%)	281 (29.02%)	119 (25.37%)

Note: *, (Number of age group/total sheep ×100%); Active cysts = Type 1 and Type 2; Calcified cysts = Type 3

### The infection rate of CE is associated with the age of sheep

**Fig. 3:**
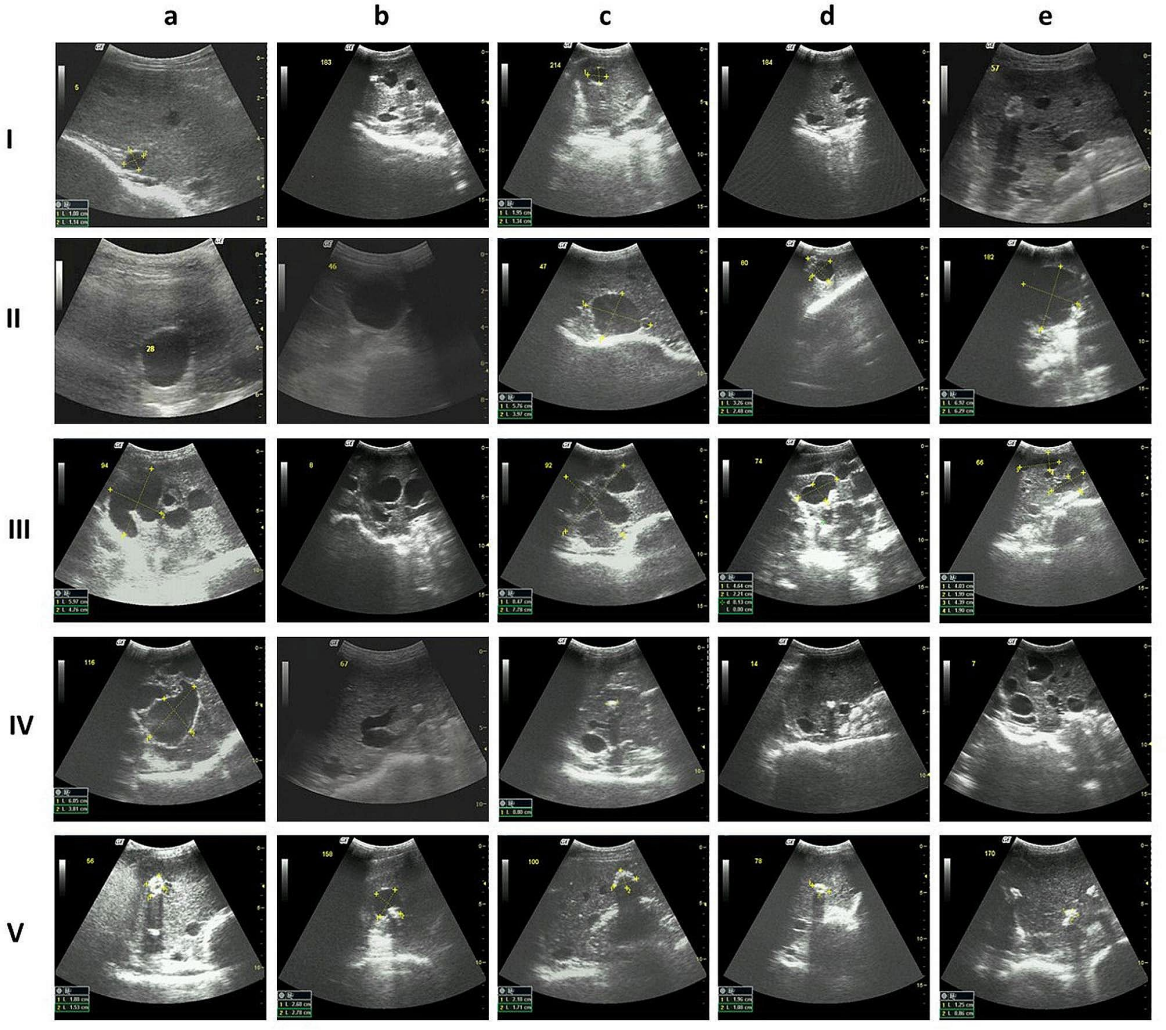
Ultrasonography detection of *Echinococcus granulosus* cysts in sheep **I**: liver with small cyst(s); **II**: liver with a single active cyst; **III**: liver with multiple active cysts; **IV**: liver with collapsed or partially calcified cysts; **V**: liver with dead or calcified cysts. a–e: Different shapes of cysts in each panel

Shaving a window area after shearing made detection easy, and clear images of the liver lesions were obtained. Using different angles and moving the detector from the left edge to the right edge of the liver in the window area through the intercostal space between the ribs achieved at least 85% detection in the liver areas. From the window area, 1–21 cysts were detected in the liver and counted, with an average of 2.12 active cysts in sheep with active cysts. Table 1 shows the overall CE prevalence of 38.43% (372/968) in the three sheep flocks in 2014. The average diameter of active cysts was 1.43 ± 0.94 cm if the sheep were infected. Infected sheep aged more than 2 years old had an average of 2.09 ± 1.07 active cysts that were 2.85 ± 2.08 cm in size. We classified sheep infected with CE into two groups based on the activity and calcification of echinococcal cysts (Table 1 and Supplementary Information 1). Figure 3I a–e show active cysts less than 1 cm in diameter, and Fig. 3II a–e show that sheep had active cysts larger than 1 cm. Figure 3III a–e show multiple unilocular cysts, indicating that one sheep’s liver contained more than one unilocular cyst. These two types of cysts are similar to CL and CE1, as classified by the WHO Informal Working Group on Echinococcosis (WHO-IWGE) for each of the multiple cysts in the liver, given that most infected sheep normally have multiple unilocular cysts in the liver. Transitional cysts comprised both active cysts and collapsed or calcified cysts, which were also marked as active cysts, and their numbers and sizes were recorded (Fig. 3IV a–e). A total of 91 sheep had active cysts, which accounted for 9.40% of the 968 sheep tested in 2014. Seventy-four sheep had active cysts, accounting for 15.78% (74/469) of all the test sheep in 2021, which is significantly higher than that in 2014 (χ^2^ = 12.642, *P* = 0.000) (Table 2). Caseated or calcified cysts, which were inactive or dead, were (s) classified as calcified cysts, which are similar to cysts classified as CE4 and CE5 by WHO-IWGE [10] (Fig. 3V a–e). A total of 281 sheep had calcified cysts, accounting for 29.02% of all checked sheep in 2014 compared to 25.37% (119/469) in 2021, which was not different from that in 2014 (χ^2^ = 1.105, *P* = 0.293). Of the sheep with active cysts, most (74.00%) had multiple unilocular cysts (Fig. 3III–IV).

**Table 2:** Comparison of infection rates of CE in the sheep in 2014 and 2021

Year	*N*	Prevalence (%)	χ^2^	*P*	active cysts (%)	χ^2^	*P*	calcified cysts (%)	χ^2^	*P*
2014	968	360 (37.19)	1.122	0.289	91 (9.40)	12.642	0.000	281(27.99)	1.105	0.293
2021	469	188 (40.09)			74 (15.78)			119 (25.37)		
Total	1437	548 (38.14)			165 (11.48)			400 (27.16)		

**Fig. 4:**
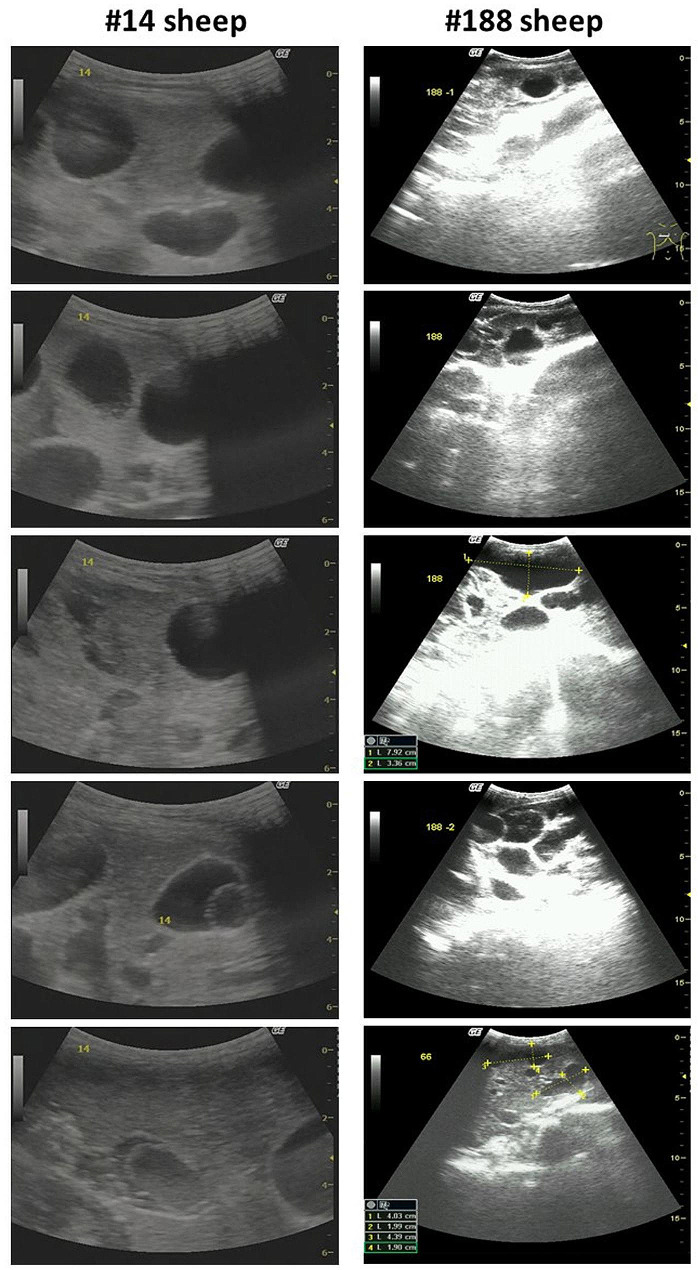
Detection of sheep liver with different scanning sections and angles of detector for cysts in two sheep

Notably, sheep may present with multiple unilocular cysts. To identify multiple cysts, we moved the detector to different positions using different angles of the detector to achieve the best detection of cysts and took 4–5 photographs to confirm the number and size of cysts (Fig. 4).

**Fig. 5:**
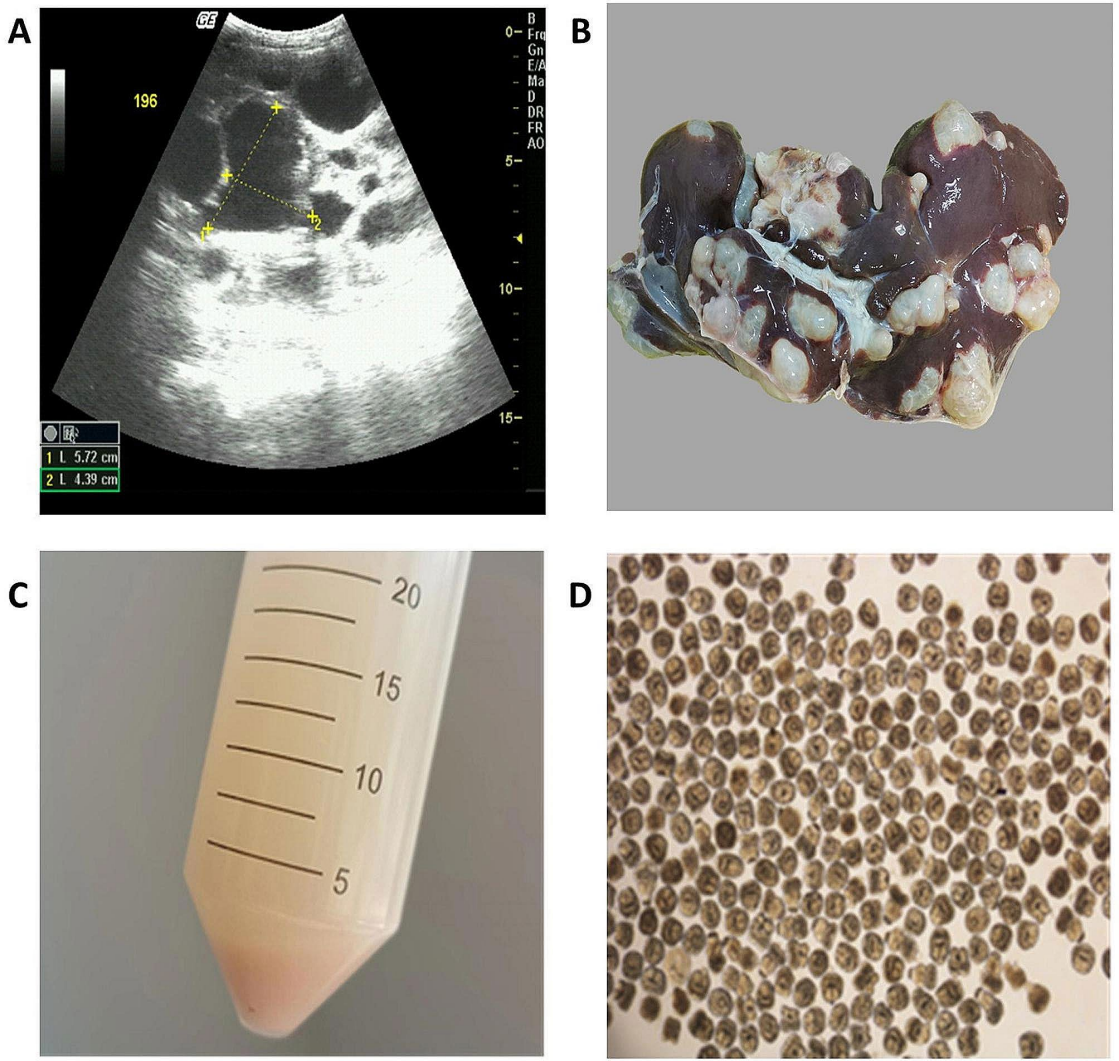
Echinococcus granulosus cysts and production of protoscoleces in dissected sheep. **A**, Ultrasonography image; **B**, Sheep liver; **C**, Volume of protoscolex precipitation; **D**, Protoscoleces under an optical microscope

The lowest infection rates of 12.50% (16/128) and 19.63% (43/219) were observed in 1-year-old and 2-year-old sheep, respectively, surprisingly, these young sheep also had a high percentage of calcified cysts 87.50% (14/16) and 95.35% (41/43) for 1- and 2-year-old sheep, respectively) among the infected sheep. Prevalence increased with age in all sheep flocks (Table 1, Tables S1–S5, Fig. 5). The Chi-square test for differences in age groups in sheep was significant (χ^2^ = 602.247, *P* < 0.01). Five-year-old sheep had the highest infection rate 59.13% (68/115), with 16.52% active cysts (Table 1). Although all of these cysts were unilocular, most infected sheep had multiple unilocular cysts in the liver, with only 36 sheep having single unilocular cysts in 2014. There was a significant difference in the infection rate between age groups (χ^2^ = 110.861, *P* < 0.01). Although 3- to > 6-year-old sheep accounted for 64.15% (621/968) of the total sheep flocks in 2014, the most active cysts 95.60%, (87/91)were from these older sheep, indicating that these sheep play a key role in the transmission of CE.

### Fertility and viability of echinococcal cysts from dissected sheep

Four sheep were purchased and slaughtered to determine the fertility of the cysts. Figure 3 shows one of the four sheep. No cysts were found in the lungs of sheep. All sheep had PSCs, and the collection of PSCs from the four sheep with fertile cysts showed 140,000–540,000 PSCs, with an average number of PSCs of 300,000. The average viability of PSCs was 89.22% (Fig. 5; Table 3). To determine the correlation between size and fertility of echinococcal cysts, we collected 122 liver cysts from 27 sheep and showed that the most fertile cysts(80%) were more than 2 cm in diameter(Supplementary Information 2).

**Table 3:** Fertility and viability of *Echinococcus granulosus* cysts in the dissected sheep

Parameter	Value
Number of sheep/Sex/Age	4/female/3,3,3,4 years old
Mean cysts (individually)	5.75 ± 5.56 (2, 3, 4, 14)
Mean fertile cysts	4.25 ± 3.86 (2, 2, 3, 10)
Percentage of fertile cysts	73.9% (17/23)
Mean precipitated PSCs	1.5 ± 0.86 (1.1, 0.7, 1.5, 2.7)
Mean number of PSCs	300,000 ± 172819.8
Viability of PSCs	89.22% (94.3%, 95.2%, 87.8%, 79.6%)

### Comparison of infection rates of CE in the sheep between 2014 and 2021

Bayingbuluke Township implemented a control program using education through posters, education teams who visited villages, and once-a-year delivery of praziquantel medicine to every household for deworming dogs as control measures. A comparison of the prevalence of sheep CE between 2014 and 2021 showed no significant difference between the infection rates in 2014 and 2021 (χ^2^ = 1.122, *P* > 0.05) (Table 2). Sheep aged 6 years and older had more than half 51.61% (16/31) of the active cysts, which was significantly higher than that in 2014.

## Discussion

Currently, serological test is available for detecting sheep CE infection [13]. The only available method for detecting sheep CE is to inspect animals in slaughterhouses. Serological diagnosis of CE infection in livestock is problematic due to the low reaction of these animals and serological cross-reactivity with several other species of taeniid cestodes, including *Taenia hydatigena* and *Taenia ovis* [14, 15]. The US technique has been widely used in clinical practice and is becoming an inexpensive tool for diagnosis. By using this technique, ante-mortem diagnosis of CE is possible [16, 17]. In this study, we showed that the US method can be used to successfully detect cystic echinococcal cysts in the livers of sheep in a remote mountain pasture area. To make this method easy to use, we opted for the US procedure using a book model to screen CE in sheep. Our group could check 100–150 sheep per day during the shearing season with the help of sheep owners. The group included four staff members during the procedure: one person shaved after shearing, one person detected, one person recorded and helped the US operator move the equipment in the field, and one person marked the examined sheep and released the animals. From the shaved or window area, approximately 80–90% of the liver can be scanned in a minute, indicating that US is a convenient, fast, and accurate method for detecting CE in sheep.

Sheep are the most adapted hosts for CE and play a key role in the dynamic transmission of this disease [18]. The liver is the organ most targeted for depositing echinococcal cysts, which is an indicator of the prevalence and, more importantly, for assessing the progress of the control program. Only PSCs play a role in transmission [1], and only active cysts, especially large active cysts, contain PSCs. Without PSCs, the host is at the dead end of its life cycle, and there will be no further transmission of this parasite. Although 38.43% of the sheep in the flocks were infected with CE in this study, we showed that only 9.40% of these sheep harbored active cysts. Our previous collection of over 100 livers from sheep aged < 2 years showed that most liver lesions were calcified, and there were no PSCs found in these young sheep (unpublished), which is similar to our finding with US. Old sheep have a higher percentage of active cysts in the liver, indicating that old sheep play an important role in transmission, given that sheep older than 5 years are the major culled aged sheep. We showed that 15.34% of these sheep had active cysts, which was 1.9-fold higher than the average (8.08%, χ^2^ = 8.912, *P* = 0.003), indicating that these sheep play an important role in the transmission of CE. If US can be used to detect infected sheep, the control program may have one more precise technical measure. In this study, we classified echinococcal cysts into two types, mainly based on their activity and calcification. Although the overall infection rate was 38.43%, only 9.40% of the sheep had active cysts. These sheep likely play a role in the transmission of echinococcosis, as only these sheep can produce PSCs. Normally, calcified cysts do not contrast PSCs and have no role in transmission.

Cyst status in the sheep liver is different from that of cysts in the human liver. Unilocular cysts are normally found in the livers of patients with CE, with very few having multiple cysts. However, sheep normally have multiple cysts in their livers. In this study, only 9.40% of sheep had active cysts, and most sheep had multiple unilocular cysts, with a maximum of 21 cysts identified in the US images. In this study, we did not find cysts containing daughter cysts in these tested sheep, indicating that very few sheep had CE2 cysts, according to the WHO IEGE cyst classification for CE patients. We believe that US is a reliable detection method for such active cysts. US may be used as a control measure to find infected sheep with fertile cysts, especially for culling older sheep as a control measure [19].

Newborn lambs aged less than 4 months normally have very small cysts when they are infected. In this study, we did not check these lambs because the cysts were too small to differentiate them from other lesions, and all of these cysts were infertile. Dueger and Gilman (2001) showed that sheep aged 1–2 years had no fertile cysts [20]. In these flocks, including newborn lambs aged less than 4 months, 47.99% of young animals aged less than 2 years were not involved in the transmission of CE.

Older sheep have more fertile cysts, and the frequency of sheep harboring at least one fertile cyst was approximately 70%, with sheep checked in Sardinian regions [21]. However, it is still unclear whether cyst size is correlated with the fertilization of echinococcal cysts. With checking 122 cysts collected from 27 sheep, we found that cysts less than 1 cm in size did not contain PSCs. We also found that most active cysts measuring more than 2 cm from sheep were fertilized. In fact, few studies have shown a correlation between cyst size and fertility, and one study showed that active cysts > 3 cm are almost fertile [22]. However, further studies are needed to determine cyst size and fertility if US is used as a control monitoring tool.

In our study, the number of infections in 2021 was higher than that in 2014. There was no significant difference between the rates (χ^2^ = 1.122, *P* > 0.05) after 7 years of dosing dogs with praziquantel by the local veterinarian, indicating that dosing dogs once a year has no effect in terms of controlling CE, given that dosing dogs eight times per year achieved progress in the control of CE in New Zealand and in other areas where similar control programs were conducted [23]. Now, two technical control measures including dosing dogs and vaccination of sheep with EG95 have been used for control of cystic echinococcosis [24, 25]. Although EG95 has not been used in the study areas, these two measures have been applied for control of echinococcosis in the high endemic areas in China, such as in Qinghai Province, which showed effective control in terms of reducing human CE cases and sheep CE prevalence [26].

## Conclusions

The US technique has been widely used in clinical practice and is becoming an inexpensive tool for diagnosis. In this study, we showed that the US method can be used to successfully detect echinococcal cysts in the livers of sheep in a remote mountain pasture area, especially to monitor the control progress at the farm scale. Our results showed that 1–2-year-old sheep play a minor role in CE transmission, as almost none of these sheep had PSCs. Old sheep, especially culled aged sheep, play a key role in the transmission of CE.

## Methods

### Study area and sheep flocks

Bayinbuluke is a township located 300 km southwest of Urumqi, the capital of Xinjiang Autonomous Region, China. The township covers 23,868 km^2^ of mountain pasture area used to raise 760,000 domestic animals by 9,945 herders in the middle of the Tianshan Mountains. In the community, the ethnic majority population is Mongolian, accounting for 92.5% of herd families. Sheep comprise the majority of livestock (90%), with approximately 10% of livestock in this community being other domestic animals, including cattle, yaks, and horses. In the shearing season (end of July), 968 ewes in three flocks in 2014 and 469 ewes in two flocks in 2021 were screened using US to probe liver CE after agreement with sheep owners. All sheep were obtained from three family-owned private farms.

### Sheep age

Sheep with only milk teeth were considered less than 1 year of age. These lambs were born in March, so by July, they were 4 months old. Sheep with one pair of permanent incisors were considered 1 year old, while 2- and 3-year-old sheep possessed two and three pairs of permanent incisors, respectively. The 4-year-old sheep had all their milk teeth replaced and possessed a full set of sharp teeth; the 5-year-old sheep had flat anterior tooth crowns with a clear gap between teeth, whereas in sheep aged 6 year-old and older, the tooth gums were sunken, and the crowns became narrow. Some teeth might have been lost.

### Screening methods

Ultrasound examination was performed with a portable real-time B-mode scanner (LOGIQ Book Model, General Electric Company, Fairfield, CT, USA) using a 3.5 MHz curved array transducer (General Electric Company, Fairfield, CT, USA) to detect sheep liver. The equipment was powered by a 3.0 kW portable electricity generator (Longpeng 3700 W, Fuan, Fujian, China). We examined sheep in the sheep-shearing season in these areas at the end of July, after milking and restocking sheep flocks. To determine the correct position for detecting liver lesions, each sheep was captured for US screening within 2–3 min without anesthesia. For the right position for screening, the sheep were placed down with the left side on the ground and the right side upward after the legs were bound. The skin area between the 9th and 13th ribs (15 cm × 15 cm) was shaved after being sheared as the “window area” for oblique plane scanning with the detector, as shown in Fig. 1. For better viewing of the screen in the open field, the equipment was placed in a shadowed box. The US coupling gel was smeared on the shaved area to allow for the transmission of ultrasound waves. The scanning operation was applied with a wide field of vision and a clear display of the shallow model. The location of the liver was first detected by probing the structures of the portal vein and hepatic duct using a clear sonogram. Ultrasound-returning echo images of the liver were obtained by selecting and adjusting the appropriate control buttons, such as gain, dynamic range, time gain compensation or depth gain compensation, frame frequency, focus number and focus depth, high resolution, and high-definition grayscale. A fixed scanning depth was used to detect and scan the entire liver. To improve image quality, we performed continuous whole liver scanning from left to right using different angles and multiple sections to scan the whole liver according to the principle of ultrasonic imaging. When abnormal echo lesions were found in the liver parenchyma, photographs were captured and saved for further evaluation and confirmation of cyst types and lesion conditions based on the internal echo and boundary of the lesions. The details of US screening for sheep liver CE are presented in Supplementary Information 1.

### Post-mortem examination

The study protocol was approved by the Medical Ethics Committee of the First Affiliated Hospital of Xinjiang Medical University (Approval No.: K1404-01) in accordance with the guidelines and regulations for laboratory animals.

Four sheep were slaughtered at local abattoirs, and their livers and lungs were examined for cysts. Echinococcal cysts were identified through surface examination and palpation of the lungs and liver post-mortem. Normal cysts are filled with clear fluid and have distinct white membranes in one or more separate chambers. For these surface cysts or cysts that can be seen, the cyst fluid was aspirated with a syringe, the PSCs were gravity-sedimented in a falcon tube and washed three times with PBS, and pieces of the cyst wall were removed with a Pasteur tube during washes. The PSCs were transferred into a 10-mL tube, and the volume of precipitated PSCs was measured after 20 min of precipitation. Number of PSCs = volume of precipitated PSCs in mL × 200,000 (note: 1 mL of precipitated PSCs contained 200,000 PSCs). The sediments from each cyst were observed under a light microscope for PSCs. The viability of the PSCs was determined for each fertile cyst by staining with 0.1% methylene blue, with dead PSCs staining blue [27]. At least 200 PSCs from each cyst were counted and characterized as dead (blue) or live (clear).

For those cysts barred in the parenchyma of the liver or lungs, parallel 1-cm slices from the parenchyma of the lungs and liver were examined. Small and completely calcified cysts that are less than 5 mm in diameter were not included in this study because it was difficult to differentiate them from other metacestode lesions.

### Statistical analysis

SPSS 22.0 was used to analyze the correlation between the age groups and the infection rate of hepatic CE. The difference in prevalence and fertility between age groups and two time points, hydatid cyst infection rates, and calcification infection rates between age groups were analyzed using the Chi-squared test, and 95% confidence intervals were computed. Statistical significance was set at *P* < 0.05.

### Electronic supplementary material

Below is the link to the electronic supplementary material.

Supplementary Material 1

Supplementary Material 2

Supplementary Material 3

Supplementary Material 4

Supplementary Material 5

Supplementary Material 6

Supplementary Material 7

## Data Availability

All data generated or analyzed during this study are included in this article and its Supplementary Information files.

## Abbreviations

CE: Cystic echinococcosis
US: Ultrasonography
WHO-IWGE: World Health Organization Informal Working Group on Echinococcosis
