# Supplementary material for: Ultrasonography screening of hepatic cystic echinococcosis in sheep flocks used for evaluating control progress in a remote mountain area of Hejing County, Xinjiang
Source: BMC Vet Res. 2024 May 17;20:207. doi: 10.1186/s12917-024-04074-z (PMC11100068; doi:10.1186/s12917-024-04074-z)
Supplement: Supplementary file 1 — Supplementary Material 1 [file 12917_2024_4074_MOESM1_ESM.doc]

**Table S1** Age groups and infectious status in flock#1 in 2014 in Bayinbuluke

| **Age** | **Number of sheep (%*)** | **Positive (%)** | **Active cysts (%)** | **Calcified (%)** |
| --- | --- | --- | --- | --- |
| 1 | 34 (9.34%) | 4 (11.76%) | 1 (2.94%) | 3 (8.82%) |
| 2 | 87 (23.90%) | 15 (17.24%) | 1 (1.15%) | 14 (16.09%) |
| 3 | 68 (18.68%) | 23 (33.82%) | 6 (8.82%) | 17 (25.00%) |
| 4 | 116 (31.87%) | 58 (50.00%) | 15 (12.93%) | 43 (37.07%) |
| 5 | 37 (10.16%) | 17 (45.94%) | 7 (18.92%) | 10 (27.03%) |
| >6 | 22 (6.04%) | 10 (45.45%) | 2 (9.09%) | 8 (36.36%) |
| Total | 364 | 127 (34.89%) | 32 (8.79%) | 95 (26.10%) |

**Note:** *, (Number of age group/total sheep ×100%); Active cysts = CL and CE1; Calcified cysts = CE4 and CE5.
