# Supplementary material for: Ultrasonography screening of hepatic cystic echinococcosis in sheep flocks used for evaluating control progress in a remote mountain area of Hejing County, Xinjiang
Source: BMC Vet Res. 2024 May 17;20:207. doi: 10.1186/s12917-024-04074-z (PMC11100068; doi:10.1186/s12917-024-04074-z)
Supplement: Supplementary file 2 — Supplementary Material 2 [file 12917_2024_4074_MOESM2_ESM.doc]

**Table S2** Age groups and infectious status in flock#2 in 2014 in Bayinbuluke

| **Age** | **Number of sheep (%*)** | **Positive (%)** | **Active cysts (%)** | **Calcified (%)** |
| --- | --- | --- | --- | --- |
| 1 | 82 (22.04%) | 9 (10.98%) | 1 (1.22%) | 8 (9.76%) |
| 2 | 74 (19.89%) | 13 (17.57%) | 1 (1.35%) | 12 (16.21%) |
| 3 | 57 (15.32%) | 26 (45.61%) | 8 (14.04%) | 18 (31.58%) |
| 4 | 90 (24.19%) | 52 (57.78%) | 12 (13.33%) | 40(44.44%) |
| 5 | 45 (12.10%) | 26 (57.78%) | 7 (15.56%) | 19 (42.22%) |
| >6 | 24 (6.45%) | 12 (50.00%) | 2 (8.33%) | 10 (41.66%) |
| Total | 372 | 138 (37.10%) | 31 (8.33%) | 107 (28.76%) |

**Note:** *, (Number of age group/total sheep ×100%); Active cysts = CL and CE1; Calcified cysts = CE4 and CE5.
