# Supplementary material for: Ultrasonography screening of hepatic cystic echinococcosis in sheep flocks used for evaluating control progress in a remote mountain area of Hejing County, Xinjiang
Source: BMC Vet Res. 2024 May 17;20:207. doi: 10.1186/s12917-024-04074-z (PMC11100068; doi:10.1186/s12917-024-04074-z)
Supplement: Supplementary file 3 — Supplementary Material 3 [file 12917_2024_4074_MOESM3_ESM.doc]

**Table S3** Age groups and infectious status in flock#3 in 2014 in Bayinbuluke

| **Age** | **Number of sheep (%*)** | **Positive (%)** | **Active cyst (%)** | **Calcified (%)** |
| --- | --- | --- | --- | --- |
| 1 | 12 (5.17%) | 3 (25.00%) | 0(0) | 3 (25.00%) |
| 2 | 58 (25.00%) | 15 (25.86%) | 0(0) | 15 (25.86%) |
| 3 | 56 (24.14%) | 21 (37.50%) | 6(10.71%) | 15 (26.79%) |
| 4 | 58 (25.00%) | 34 (58.62%) | 13 (22.41%) | 21 (36.21%) |
| 5 | 33 (14.22%) | 25 (75.76%) | 5(15.15%) | 20 (60.60%) |
| >6 | 15 (6.47%) | 9 (60.00%) | 4(26.67%) | 5 (33.33%) |
| Total | 232 | 107 (46.12%) | 28 (12.07%) | 79 (34.05%) |

**Note:** *, (Number of age group/total sheep ×100%); Active cysts = CL and CE1; Calcified cysts = CE4 and CE5.
