# Supplementary material for: Ultrasonography screening of hepatic cystic echinococcosis in sheep flocks used for evaluating control progress in a remote mountain area of Hejing County, Xinjiang
Source: BMC Vet Res. 2024 May 17;20:207. doi: 10.1186/s12917-024-04074-z (PMC11100068; doi:10.1186/s12917-024-04074-z)
Supplement: Supplementary file 4 — Supplementary Material 4 [file 12917_2024_4074_MOESM4_ESM.doc]

**Table S4** Age groups and infectious status in flock#1 in 2021 in Bayinbuluke

| **Age** | **Number of sheep (%*)** | **Positive (%)** | **Active cysts (%)** | **Calcified (%)** |
| --- | --- | --- | --- | --- |
| 1 | 19 (7.22%) | 6 (31.58%) | 3 (15.79%) | 3 (15.79%) |
| 2 | 23 (8.75%) | 3 (13.04%) | 2 (8.70%) | 1 (4.35%) |
| 3 | 45 (17.11%) | 18 (40.00%) | 6 (13.33%) | 12 (26.67%) |
| 4 | 90 (34.22%) | 51 (56.67%) | 14 (15.56%) | 37 (41.11%) |
| 5 | 61 (23.19%) | 32 (52.46%) | 15 (24.59%) | 17 (27.87%) |
| >6 | 25 (9.51%) | 16 (64.00%) | 11 (44.00%) | 5 (20.00%) |
| Total | 263 | 126 (47.91%) | 51 (19.39%) | 75 (28.52%) |

**Note:** *, (Number of age group/total sheep ×100%); Active cysts = CL and CE1; Calcified cysts = CE4 and CE5.
