# Supplementary material for: Ultrasonography screening of hepatic cystic echinococcosis in sheep flocks used for evaluating control progress in a remote mountain area of Hejing County, Xinjiang
Source: BMC Vet Res. 2024 May 17;20:207. doi: 10.1186/s12917-024-04074-z (PMC11100068; doi:10.1186/s12917-024-04074-z)
Supplement: Supplementary file 5 — Supplementary Material 5 [file 12917_2024_4074_MOESM5_ESM.doc]

**Table S5** Age groups and infectious status in flock#3 in 2021 in Bayinbuluke

| **Age** | **Number of sheep (%*)** | **Positive (%)** | **Active cysts (%)** | **Calcified (%)** |
| --- | --- | --- | --- | --- |
| 1 | 2 (0.97%) | 0 (0) | 0 (0) | 0 (0) |
| 2 | 43 (20.87%) | 5 (11.63%) | 2 (4.65%) | 3 (6.98%) |
| 3 | 10 (49.03%) | 28 (27.72%) | 6 (5.94%) | 22 (21.78%) |
| 4 | 45 (21.84%) | 21 (46.67%) | 5 (11.11%) | 16 (35.56%) |
| 5 | 9 (4.37%) | 7 (77.78%) | 5 (55.56%) | 2 (22.22%) |
| >6 | 6 (2.91%) | 6 (100.00%) | 5 (83.33%) | 1 (16.67%) |
| Total | 206 | 67 (32.52%) | 23 (11.17%) | 44 (21.36%) |

**Note:** *, (Number of age group/total sheep ×100%); Active cysts = CL and CE1; Calcified cysts = CE4 and CE5.
