# Supplementary material for: Ultrasonography screening of hepatic cystic echinococcosis in sheep flocks used for evaluating control progress in a remote mountain area of Hejing County, Xinjiang
Source: BMC Vet Res. 2024 May 17;20:207. doi: 10.1186/s12917-024-04074-z (PMC11100068; doi:10.1186/s12917-024-04074-z)
Supplement: Supplementary file 6 — Supplementary Material 6 [file 12917_2024_4074_MOESM6_ESM.doc]

**Supplementary Information 1**

**Procedure and tips for scanning sheep cystic echinococcosis using the B-mode book model ultrasonography scanner**

1. Mountainous areas normally have wet ground. Therefore, the ground of the test area close to the sheep flocks was covered with a plastic film sized 4 × 8 m to protect the area from the muddy condition.

2. The electronic detector powered by a portable electricity generator (1.5–3.0 kW) was started after setting all the wires and sockets ready at the testing ground.

3. The sheep was tied up and sheared.

4. A window area was shaved after shearing the sheep. To find the right position for detecting liver cysts, each sheep was laid down with the left side on the ground and the right side upward after the legs were bound. A skin area between the 9th and 13th ribs sized 15 × 15 cm was shaved with an electronic shaver as the window area after shearing, which is the right position for detecting the liver as indicated in Figure 1.

5. Ultrasonography coupling gel was smeared on the shaved area to allow a good transmission of ultrasound waves.

6. Ultrasound examination was performed with a real-time B-mode scanner. A veterinary animal abdominal ultrasonography scanner or any similar model of a US scanner with a 3.5-MHz curved array transducer or similar model of transducer was applied.

7. When abnormal echo lesions were found in the liver parenchyma, photo images were snapped and saved for further evaluation and confirmation of cyst types and lesion conditions based on the internal echo and boundary of the lesions.

8. The scanning operation was conducted, with a broad vision and clear display of the shallow model. First, the coupling agent was smeared evenly on the surface of the 3.5-MHz convex array ultrasonic detector, and placed on the skin window area. The portal vein and hepatic duct were then identified using the anatomical position of the hepatic portal and color doppler technology. This allowed the determination of the position of the liver, and then the whole liver was scanned using different angles or by moving the detector in the window area from the left edge of the liver to the right edge of the liver. Notably, the structure of the portal vein and hepatic duct was used to determine the right position of the liver.

9. Based on the size and depth of the liver and lesion location, the appropriate control buttons, such as gain, dynamic range, time gain compensation or depth gain compensation, frame rate, number and depth of focus, high-resolution, and high-definition grayscale, were selected and adjusted to obtain clear ultrasonic echo images. For lesions in deep positions, the resolution was reduced to increase the clarity of the deep lesion. For lesions in shallow positions, the resolution, focus position, and the number of focuses were increased accordingly so that the internal and boundary of the lesions can be more clearly displayed.

10. To improve the image quality using the ultrasonic imaging principle, different angles and multiple sections of lateral dynamic ultrasound probes were used by moving the detector from the left edge to the right edge of the liver through the gap between the ribs to scan the maximum area of the liver. This way, at least 85% of the whole liver area was detected through the intercostal space between the ribs in the window area.

11. When abnormal echo lesions are found in the liver parenchyma during scanning, longitudinal and transverse sections are required to determine the internal echo and boundary or edge of the lesions, and to determine the cyst type or other lesions. Next, snap and save the ultrasonic image for further evaluation and confirmation (NB: Avoid artificial disturbance by moving probe and sheep movement. When detecting abnormal echoes as lesions, images should be frozen and saved, and each image should be given a digital marker or name. If recording video, the images should be saved in the book B-mode scanner and transferred to a USB flash drive).

12. Classification of cystic echinococcosis and these liver echinococcal cysts into three categories:

- Figures 3I a–e: active cysts with cyst diameter of less than 1 cm
- Figures 3II a–e: active cysts with cyst size (diameter) of more than 1 cm
- Figures 3III a–e:multiple unilocular cysts ,indicating that one sheep’s liver contained more than one unilocular cyst. These two types of cysts are similar to CL, CE1, as classified by the WHO Informal Working Group on Echinococcosis (WHO-IWGE) for each of the multiple cysts in the liver, given that most infected sheep normally have multiple cysts in the liver
- Figures 3IV a–e: Transitional cysts comprised both active cysts and collapsed or calcified cysts, which were also marked as active cysts, and their numbers and size were recorded
- Figures 3V a–e: Caseated or calcified cysts ,which were inactive or dead, were(s) classified as calcified cysts ,which are similar to cysts classified as CE4 and CE5 by WHO-IWGE.
- Notably, if a sheep harbored multiple cysts, and contained cysts larger than 1 cm. To identify the multiple cysts, we moved the detector to different positions using different angles of the detector to obtain the best detection of cysts and took four to five pictures to confirm the number and size of the cysts (Figure 4).

**Reference**

1. WHO Informal Working Group**: International classification of ultrasound images in cystic echinococcosis for application in clinical and field epidemiological settin**gs*. Acta tropic*a 2003**,** 85(2):253-261.
