# Supplementary figures and images for: Ultrasonography screening of hepatic cystic echinococcosis in sheep flocks used for evaluating control progress in a remote mountain area of Hejing County, Xinjiang
Source: BMC Vet Res. 2024 May 17;20:207. doi: 10.1186/s12917-024-04074-z (PMC11100068; doi:10.1186/s12917-024-04074-z)

## Slide 1
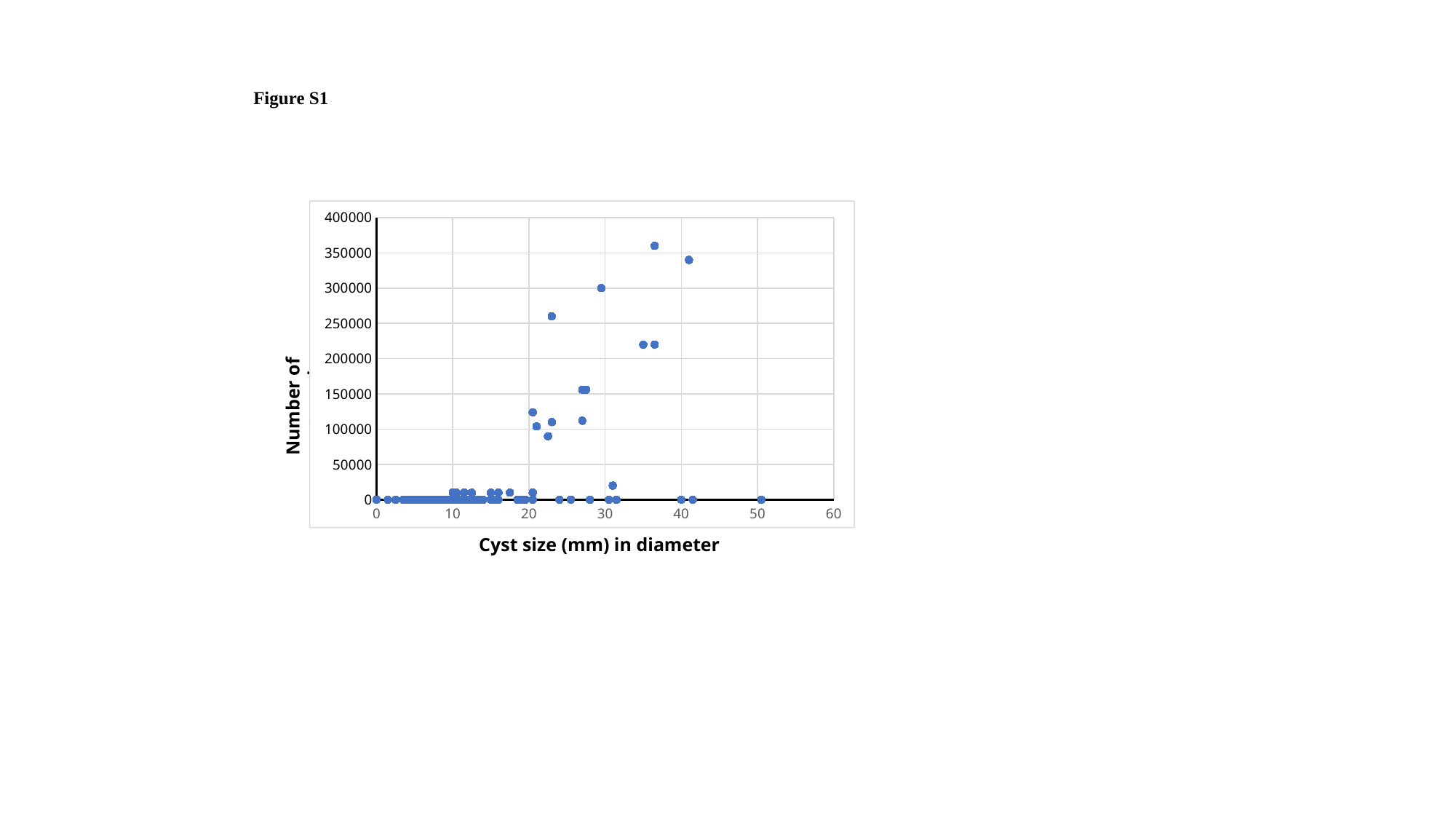

Figure S1
### Chart
| Category | 原头蚴数 |
|---|---|Number of protoscoleces
Cyst size (mm) in diameter

Supplement: Supplementary file 7 — Supplementary Material 7 [file 12917_2024_4074_MOESM7_ESM.pptx]
